# ST-Segment Elevation Myocardial Infarction (STEMI) With a Fishy Twist: A Case of Kounis Syndrome Following Seafood Ingestion

**DOI:** 10.7759/cureus.113608

**Published:** 2026-07-29

**Authors:** Sophie R Fisher, Aiden M Van Loo, Athanasios Rempakos, Steven B Timmis

**Affiliations:** 1 Medicine, Oakland University William Beaumont School of Medicine, Rochester, USA; 2 Internal Medicine, Corewell Health William Beaumont University Hospital, Royal Oak, USA; 3 Cardiovascular Medicine, Corewell Health William Beaumont University Hospital, Royal Oak, USA; 4 Cardiology, Beaumont Health, Royal Oak, USA

**Keywords:** allergy and anaphylaxis, kounis case study, kounis syndrome (ks), mi kounis, myocardial infarction, percutaneous coronary intervention, st-elevation myocardial infarction (stemi), st-segment elevation myocardial infarction (stemi), type2 kounis

## Abstract

Kounis syndrome is an underrecognized condition characterized by the concurrence of acute coronary syndrome (ACS) and a hypersensitivity reaction mediated by mast cell activation. Diagnosis can be challenging, and treatments for anaphylaxis and ACS may have competing effects. We report a case of a 58-year-old male who presented with anaphylaxis after seafood ingestion, complicated by anterior ST-segment elevation myocardial infarction (STEMI). Coronary angiography revealed plaque rupture with thrombus in the mid-left anterior descending artery, consistent with type II Kounis syndrome. This case highlights the importance of early recognition and management to balance treatment of both allergic and ischemic processes.

## Introduction

Anaphylaxis is a severe, life-threatening, systemic hypersensitivity reaction characterized by rapid onset of airway compromise, hypotension, and end-organ dysfunction [1]. Prompt administration of epinephrine is the cornerstone of therapy and is critical in reducing morbidity and mortality. In rare cases, the hypersensitivity reaction can trigger an acute coronary syndrome (ACS), termed Kounis syndrome (KS) [2]. Based on pathophysiology, KS is classified into three subtypes: type I, involving coronary vasospasm in patients without underlying coronary disease; type II, involving coronary vasospasm with plaque rupture and thrombosis in patients with preexisting atherosclerosis; and type III, involving stent thrombosis. We present a case of type II KS that occurred in the setting of an anaphylactic reaction after seafood ingestion.

This case was previously presented as a poster at the 2026 American College of Cardiology Annual Scientific Session, March 28-30, 2026.

## Case presentation

**Figure 1:**
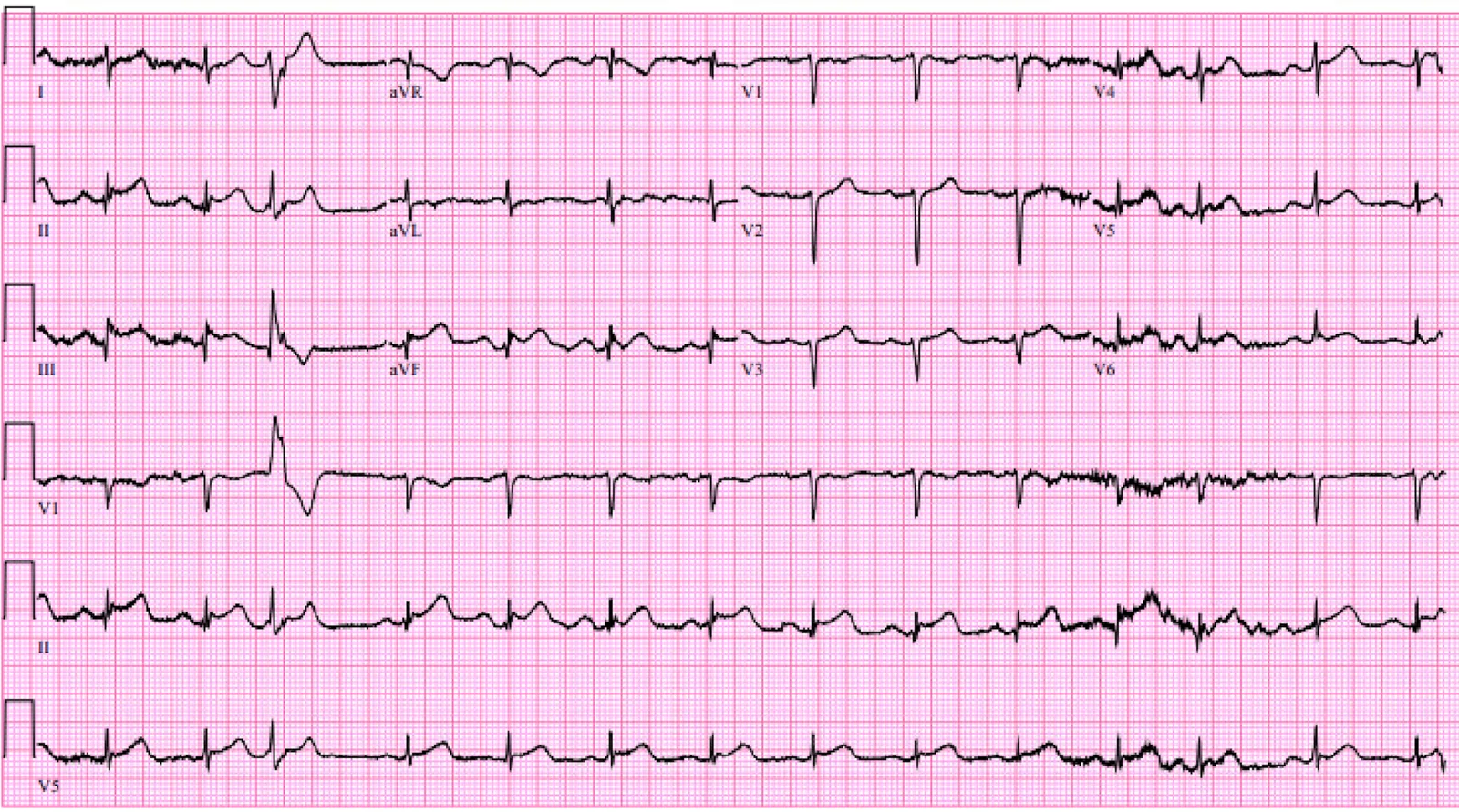
ECG demonstrating ST-segment elevations in the anterolateral (V4-V6) and inferior (II, III, aVF) leads

A 58-year-old male with a history of hypertension, hyperlipidemia, type 2 diabetes mellitus, and obesity presented with acute-onset angioedema, hypotension, and respiratory distress shortly after seafood ingestion. Emergency medical services administered intramuscular epinephrine (0.2 mg x3). In the emergency department, the patient had persistent hypotension, requiring an epinephrine infusion. An electrocardiogram (ECG) demonstrated ST-segment elevation in the anterolateral and inferior leads, concerning for transmural myocardial ischemia (Figure 1).

**Figure 2:**
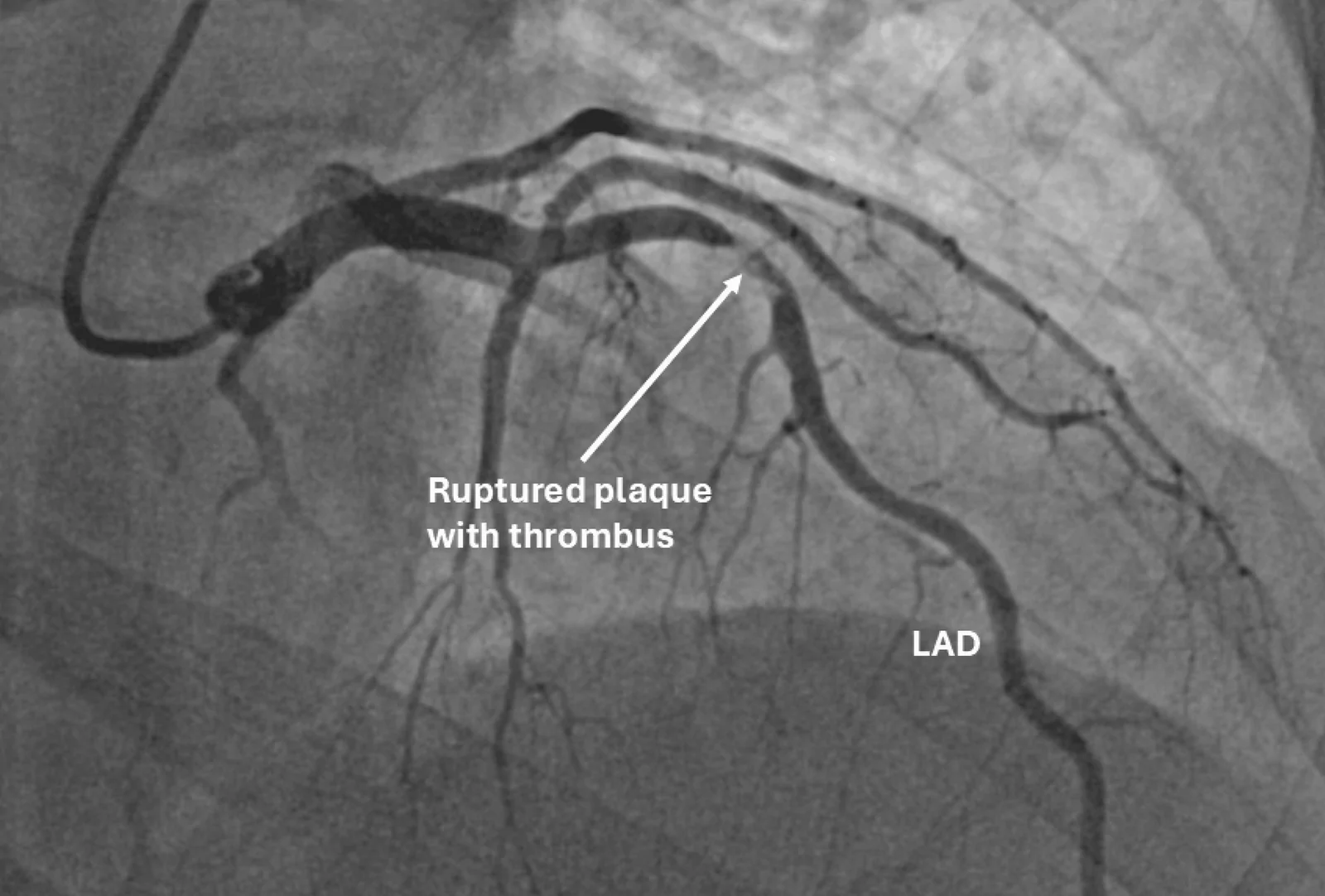
Angiogram of the left anterior descending coronary artery

Although the patient denied chest pain, he was referred for emergent coronary angiography given persistent ST-segment changes. Invasive angiography demonstrated 95% stenosis of the mid-left anterior descending (LAD) coronary artery due to plaque rupture with superimposed thrombus (Figure 2).

**Figure 3:**
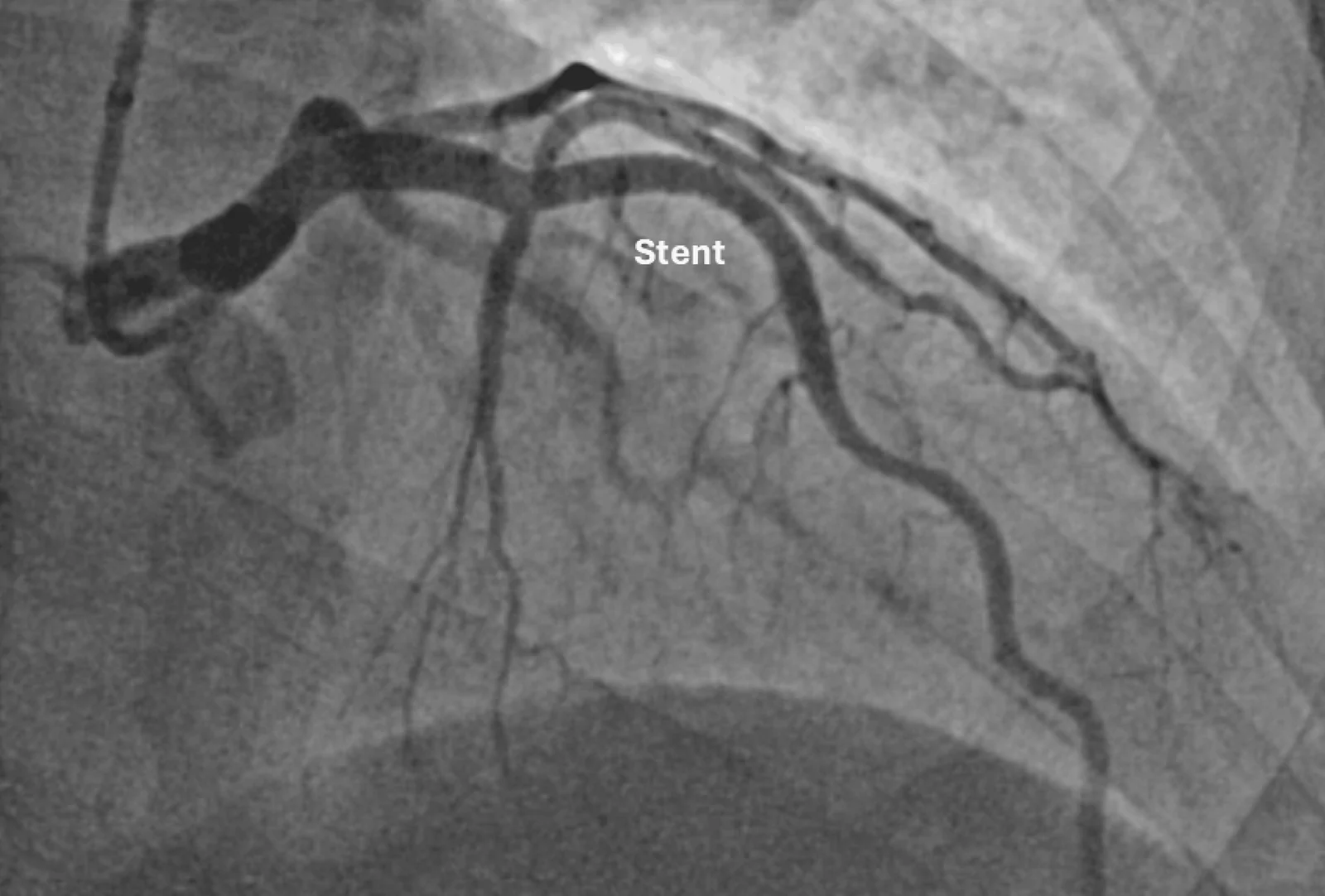
Angiogram of the left anterior descending coronary artery after stent implantation

The circumflex and right coronary arteries had mild atherosclerotic disease. A drug-eluting stent was deployed in the mid-LAD with restoration of thrombolysis in myocardial infarction grade 3 (TIMI-3) flow (Figure 3).

Following revascularization, the patient had resolution of the ST-segment elevation and was hemodynamically stable. His left ventricular function remained normal with an ejection fraction of 65%. He was discharged on hospital day 3 on dual antiplatelet therapy, high-intensity statin, ezetimibe, carvedilol, losartan, empagliflozin, corticosteroids, and antihistamines.

## Discussion

Kounis syndrome, characterized by the concurrence of an acute allergic reaction and acute coronary syndrome, represents the intersection of allergic and cardiovascular pathophysiology [2,3]. The underlying mechanism is driven by mast cell and basophil activation in response to an allergen, leading to release of inflammatory mediators, including histamine, leukotrienes, prostaglandins, tryptase, and chymase [4,5]. These mediators can induce coronary artery spasm, endothelial dysfunction, and platelet aggregation. Additionally, proteolytic enzymes activate matrix metalloproteinases, contributing to degradation of the fibrous cap and precipitating plaque rupture and thrombosis [3]. This mechanism is particularly relevant in patients with underlying atherosclerotic disease, as we observed in this case.

Diagnosis can be challenging and requires a high index of suspicion. Notably, our patient denied chest pain despite clear evidence of ST-segment elevation on the ECG, highlighting the fact that patients with KS may not present with classic ischemic symptoms. Early recognition relies on identifying the temporal association between allergic exposure and abnormal cardiac findings, such as chest pain, arrhythmia, or persistent hypotension.

Management presents a therapeutic dilemma, as treatments for anaphylaxis and ACS may have competing effects. Although epinephrine is the first-line therapy for anaphylaxis and was appropriately administered in this case, it can increase myocardial oxygen demand and exacerbate coronary vasospasm, which may worsen myocardial ischemia [3]. However, it remains essential in life-threatening anaphylaxis and should not be withheld. Conversely, beta-blockers may worsen anaphylaxis by reducing responsiveness to epinephrine and allowing unopposed alpha-adrenergic activity [6]. Morphine, commonly used in ACS, can promote mast cell degranulation and potentially exacerbate the allergic response.

Optimal management requires a balanced approach that addresses both the hypersensitivity reaction and myocardial ischemia. In addition to standard ACS therapies such as revascularization, corticosteroids and antihistamines should be administered to mitigate the allergic response [3]. Although corticosteroids are typically contraindicated in acute myocardial infarction because of concerns for impaired myocardial healing, they are indicated in KS to treat the underlying allergic inflammatory process. Vasodilators, including nitrates and calcium channel blockers, may also be beneficial in reducing coronary vasospasm. Early recognition is critical for guiding appropriate therapy and improving outcomes.

## Conclusions

This case underscores the importance of considering Kounis syndrome in patients presenting with an allergic reaction and abnormal cardiovascular findings, even in the absence of chest pain. Prompt diagnosis allows clinicians to tailor management strategies that address both pathophysiologic processes while minimizing potential harm.
